# A Multiatlas Segmentation Using Graph Cuts with Applications to Liver Segmentation in CT Scans

**DOI:** 10.1155/2014/182909

**Published:** 2014-09-08

**Authors:** Carlos Platero, M. Carmen Tobar

**Affiliations:** Department of Computer Sciences, ETSIDI, Technical University of Madrid, Ronda de Valencia 3, 28012 Madrid, Spain

## Abstract

An atlas-based segmentation approach is presented that combines low-level operations, an affine probabilistic atlas, and a multiatlas-based segmentation. The proposed combination provides highly accurate segmentation due to registrations and atlas selections based on the regions of interest (ROIs) and coarse segmentations. Our approach shares the following common elements between the probabilistic atlas and multiatlas segmentation: (a) the spatial normalisation and (b) the segmentation method, which is based on minimising a discrete energy function using graph cuts. The method is evaluated for the segmentation of the liver in computed tomography (CT) images. Low-level operations define a ROI around the liver from an abdominal CT. We generate a probabilistic atlas using an affine registration based on geometry moments from manually labelled data. Next, a coarse segmentation of the liver is obtained from the probabilistic atlas with low computational effort. Then, a multiatlas segmentation approach improves the accuracy of the segmentation. Both the atlas selections and the nonrigid registrations of the multiatlas approach use a binary mask defined by coarse segmentation. We experimentally demonstrate that this approach performs better than atlas selections and nonrigid registrations in the entire ROI. The segmentation results are comparable to those obtained by human experts and to other recently published results.

## 1. Introduction

Segmentation of 3D CT images of the liver is generally the first step in computer-assisted diagnosis and surgery systems for liver diseases. Segmentation of the liver in such scans is a challenging task due to the large anatomical variability among patients. Some overviews on the segmentation of CT liver scans are given in [1, 2]. These methods have been classified into two categories: data-driven approaches and model-based approaches. The first group is based on grey-level intensities, such as thresholding, clustering, or region growing. Their major drawback is the adjacent organ separations (e.g., stomach, kidney, and heart), which may have intensities similar to that of the liver. For these images, automatic segmentation of the liver based on the grey value alone is almost infeasible. The relation between pixel intensities and their assigned labels is generally weak. For example, region-growing approaches leak into surrounding tissue and require subsequent manual corrections [3, 4]. For such images, the intensity alone is insufficient for obtaining a robust segmentation. These cases demand the incorporation of prior knowledge of the structures to be segmented. The second group, model-based segmentation, is a global approach that matches a prior model with given data. This approach is usually based on a geometrical or statistical model. Normally, constructing a good prior shape model is difficult due to the large interpatient variability. The statistical shape model is frequently used for liver segmentation because of its ability to constrain the segmentation to match shapes observed in a training database [5, 6]. In many cases, these approaches do not make full use of the appearance. To overcome this drawback, the level set-based variational approaches allow the incorporation of prior shapes into edge-based and region-based models [7, 8].

A popular method for incorporating prior information in the segmentation process is atlas-based segmentation. In this paper, following Aljabar et al. [9], we consider that an atlas is an image in one modality with its respective labelling (which are often generated by manual segmentation). Atlas-based methods start by registering an atlas image into the target image (usually with an intensity-based similarity measure). The resulting transformation is then used to deform the atlas-labelled image into the domain of the target image. This process is often called label propagation. These approaches have a simple process compared to other generic segmentation techniques: only a registration method and an atlas are required. However, segmentations with a single atlas are intrinsically biased towards the shape and the appearance of a subject [10]. Several studies have shown that approaches that incorporate the properties of a group of atlases outperform the use of a single atlas [11, 12].

On the other hand, the main source of error in these approaches depends on the registration techniques. Indeed, insufficient similarity between the registered atlas and the target image often produces unreliable segmentations. An improvement could be achieved by registering the atlases only near the object of interest and not in the entire image [13]. Therefore, we propose a process for refining the segmentation. An initial solution is obtained using low-level operations that define the regions of interest (ROIs). Then, a combination of strategies based on a group of atlases can be applied for each ROI, potentially making the registration and segmentation operations more successful. Liver segmentation from CT scans is a good example of these difficulties in registration-based approaches. The success of a registration in an abdominal CT is compromised by the complexity of this scenario. Before performing any registration, the ROI around the liver needs to be defined.

There are two different atlas-based segmentation strategies that use a group of atlases: (a) probabilistic atlas and (b) multiatlas segmentation. We discuss the advantages and disadvantages of these two atlas-based segmentation strategies with the use of multiple atlases and how they can be combined.

*Probabilistic Atlas*. In general, a probabilistic atlas is a spatial statistical model of the appearance and shape of some structures to be studied. The construction of a probabilistic atlas consists of a spatial-normalisation step as well as an intensity-normalisation step. The spatial normalisation is needed to capture the intersubject variability of the structures. Spatial normalisation of the training atlases can be achieved with different registration algorithms. Because registration methods are a trade-off between warp regularisation and the fidelity term, probabilistic atlases possess arbitrary sharpness: weak regularisation leads to a sharp atlas, whereas strong regularisation yields a blurry atlas [14]. Given a smoothness parameter that controls the registration, an iterative atlas generation scheme is usually employed [11, 15, 16].

Subsequently, the statistical parameter spatial maps, which belong to the probabilistic atlas, are computed for each label. The simplest probabilistic atlas provides only the prior probability of labels at a spatial position and no information regarding the expected appearance of the image [15, 16]. More complex probabilistic atlases provide statistics on the relationship between the labels and the intensities [17, 18]. We focus on a probabilistic atlas by modelling both the appearance and shape of the objects to be segmented.

Once the probabilistic atlas is constructed and given an image to be segmented, the probabilistic atlas is registered into the target image, and then it is used in the segmentation task as prior information in the Bayesian formulation [15–17]. The advantage of this approach is that once the probabilistic atlas has been generated, only a single registration from the atlas to the target image is required to obtain a segmentation. However, this method depends on the success of a single registration. To overcome this drawback, the new approaches combine the registration and segmentation of a unseen image as an iterative process of estimating the labelling and calculating of the registration parameters [14, 19–21]. In this work, we construct a probabilistic atlas, and a coarse segmentation of the target image is obtained by applying the probabilistic atlas using an iterative process of affine registration and segmentation.

*Multiatlas Segmentation*. Atlases within a database can be registered to a target image, and their segmentations can be transformed and subsequently fused to provide a consensus segmentation. The main benefit of the multiatlas segmentation approach is that the effect of errors associated with any single atlas propagation can be reduced in the process of combination. The main drawback is the computational complexity. Indeed, the computational time for segmentation increases linearly with the number of atlases that have to be registered. Some authors [9, 12] have demonstrated that the precision in segmentation is improved as more atlases are combined. The overlap accuracy of a multiatlas segmentation starts to rapidly increase and then very slowly increases as more segmentations are fused. Therefore, an atlas selection is required such that the number of atlases is as low as possible so that no further improvement is expected when more atlases are added. Finally, a label fusion method is also required to obtain a consensus segmentation. The fusion of the propagated segmentations can be achieved in different ways: majority voting rule [11], STAPLE [22], or minimisation of an energy function with intensity and prior terms [23]. Therefore, the nonrigid registrations, the atlas selection, and the label fusion method must be investigated to improve the performance of the multiatlas segmentation approach.

This paper is organised as follows. In Section 2, the proposed method of combining low-level operations with a probabilistic atlas and a multiatlas segmentation approach is presented. The experiments that are performed for the liver segmentation are described in Section 3. The results and conclusions are presented in Section 4.

## 2. Methods

**Figure 1:**
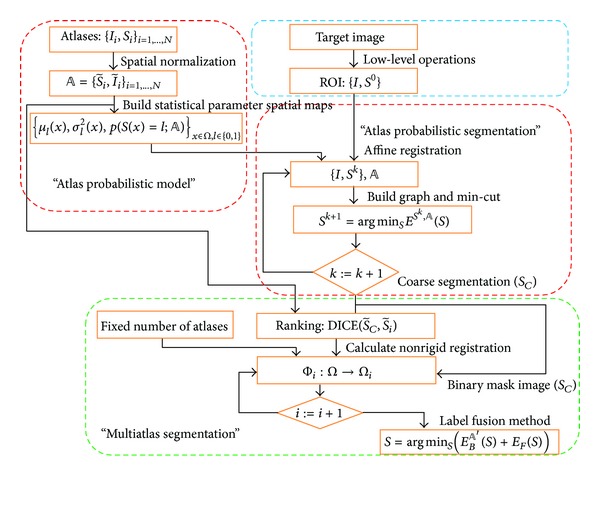
Flow chart summarising the three main steps of the proposed method.

A flow chart of the proposed framework is shown in Figure 1. Given an initial solution of the object of interest by using low-level operations, a ROI is determined. Next, a fast probabilistic atlas is applied to the ROI, and a coarse binary segmentation (*S*_*C*_) is calculated using an iterative process of segmentation-affine registration. *S*_*C*_ is a binary mask image, which is used to define the domain of nonrigid registrations and similarity measures of the atlas selection. Finally, the atlases are ranked and the selected atlases are propagated to the target image, and a label fusion method, which is based on minimisation of a discrete energy function, improves the segmentation with higher accuracy. Below, we present how the ROI of the liver is obtained, the segmentation method and the combination of a probabilistic atlas with a multiatlas segmentation.

### 2.1. Initial Solution

An initial solution is required to define the region of interest around the anatomical structure to be segmented. The initial solution allows us to introduce prior knowledge regarding the segmentation problem using low-level operations. For example, in the case of liver segmentation from these CT scans, the liver and heart have nearly the same intensity, and a liver-heart separation algorithm has to be applied to prevent oversegmentation [3]. In particular, a combination of conventional and specific techniques is applied to obtain the initial solution: liver-heart separation, nonlinear diffusion, 3D edge detection, and morphological postprocessing. First, a liver-heart separation surface is computed following [3]. The segmentations of the lungs are calculated. For each coronal slice, a minimal length curve is found, which connects the bottom of the right lung lobe with the bottom of the left lung lobe. The set of these curves defines the liver-heart separation surface. Then, the target image is filtered using a nonlinear diffusion filter with selection of the stopping time [24]. The filtered image is similar to a piecewise smooth model. Histogram analysis, 3D edge detection, and the liver-heart separation surface are applied to the filtered image, which produces a partition in isolated regions. The segmentation is followed by various postprocessing steps in which the size (largest organ) and the location (mostly on the right side) are used to determinate the initial solution. We denote this initial segmentation as *S* ^0^.

### 2.2. Segmentation Method

Both the probabilistic atlas and the multiatlas segmentation run a segmentation method based on minimising a pseudo-Boolean function by using a graph-cut technique. A conditional random field (CRF) model [25] is used to incorporate terms for appearance and shape, which are estimated from the training atlases. Other authors have previously used this framework [23, 26, 27]. Our method incorporates the following differences: (a) a generative appearance model based on the intensity from each pixel and its neighborhood, (b) a label prior probability which is estimated using a majority voting rule, and (c) a spatial regulariser that minimises the surface of separation between two different labels using a Finsler metric [28].

Consider a set of *N* training atlases for a ROI with binary labelling {*A*_*i*_}_*i*=1,…,*N*_ = {*I*_*i*_, *S*_*i*_}_*i*=1,…,*N*_ and a target image *I* with initial solution *S* ^0^, where *I*_*i*_ : *Ω*_*i*_ ⊂ *R* ^*n*^ → *R*, *n* = 3, and *S*_*i*_ : *Ω*_*i*_ ⊂ *R* ^*n*^ → {0,1} are the labelled maps. In the labelled images, object pixels are labelled as *S*(*x*) = 1 background pixels as *S*(*x*) = 0. Let Φ_*i*_ : *Ω* → *Ω*_*i*_ be the spatial mapping from the target image coordinates to the coordinates of the *i*th training subject. For simplicity, we assume that {Φ_*i*_}_*i*=1,…,*N*_ have been precomputed using a pairwise registration procedure. This assumption allows us to shorthand $A={\left\{{\tilde{S}}_{i}=S_{i}\circ \Phi_{i},{\tilde{I}}_{i}=I_{i}\circ \Phi_{i}\right\}}_{i=1,\ldots ,N}$ as the training set into the common coordinates.

The segmentation of an image *I*, based on image intensities and prior knowledge, is computed by the minimisation of a discrete energy function:

(1) S∗=argmin⁡SEA(S),  EA(S)=EBA(S)+EF(S),

where the term *E*_*B*_^*A*^(*S*) is derived from *A* using the framework of Bayesian estimation theory and *E*_*F*_(*S*) is associated with an image-based Finsler metric. We consider *S* to be a discrete random field with a neighbourhood system *E*, which is the set of edges connecting variables in the random field. The CRF model is defined by unary and pairwise potentials:

(2) E(S)=∑x∈Ωψx(S(x);I,A)+∑x,y∈Eψxy(S(x),S(y);I).

The unary potentials of the CRF model are defined as the negative log of the likelihood of a label being assigned to a pixel. It is computed from an appearance model and a label prior. The pairwise edge potentials of the CRF take the form of a contrast-sensitive Potts model.

*The Unary Potentials*. The Bayesian formulation allows the incorporation of prior information about the shape and appearance of the structures to be segmented. To find the maximum, a posteriori probability (MAP) estimation is equivalent to minimising the following energy function where the Bayes theorem is applied:

(3) $$\begin{array}{c} E_{B}^{A}\left(S\right)=-\log\left(p\left(S\;|\;I;A\right)\right)=-\log\left(\frac{p\left(I\;|\;S;A\right)p\left(S;A\right)}{p\left(I;A\right)}\right). \end{array}$$

We assume that the observed intensities of *I* are independent random variables. The image likelihood *p*(*I*∣*S*; *A*, *S* ^*k*^) can then be written as a product of the likelihoods of the individual pixels

(4) p(I ∣ S;A)=∏x∈Ωp(I(x) ∣ S(x);A).

In general, the intensity distribution is modelled by a mixture of Gaussians [19, 20]. Alternatively, we use a Gaussian distribution for each pixel and for each label [18, 21]:

(5) p(I(x) ∣ S(x)=l;A) =exp⁡(−(I(x)−μl(x))2/2σl2(x))/σl(x)∑j∈{0,1}exp⁡(−(I(x)−μj(x))2/2σj2(x))/σj(x),

where *l* ∈ {0,1}. We estimate the statistical parameters *μ*_*l*_(*x*) and *σ*_*l*_^2^(*x*) from the registered atlases as follows:

(6) μl(x)=∑i∈Ql(x)I~i(x)#Ql(x),σl2(x)=∑i∈Ql(x)(I~i(x)−μl(x))2#Ql(x)−1,

where $Q_{l}(x)=\{i|{\tilde{S}}_{i}(x)=l\}$. The means and variances are estimated using a variable number of samples #*Q*_*l*_(*x*). To overcome bias in the statistical maps, they are smoothed by means of linear diffusion with Neumann boundary conditions:

(7) μl(x)=hσs(x)∗∑i∈Ql(x)I~i(x)#Ql(x),σl2(x)=hσs(x)∗∑i∈Ql(x)(I~i(x)−μl(x))2#Ql(x)−1,

where ∗ denotes the convolution operator and *h*_*σ*_*s*__(*x*) is a Gaussian mask, with *σ*_*s*_ as the scale parameter [29], which is a tunable parameter.

The label prior probability *p*(*S*; *A*) models the joint probability of all pixels in a particular label configuration. Instead, we assume that the prior probability that a pixel *x* has label *l* only depends on its position:

(8) p(S;A)=∏x∈Ωp(S(x);A).

For each pixel *x* and each label *l* ∈ {0,1}, we define

(9) p(S(x)=l;A)=#Ql(x)N.

The image likelihood and label prior terms are combined to define the unary potentials *ψ*_*x*_(*S*(*x*); *I*, *A*):

(10) ψx(S(x);I,A) =−log⁡(p(I(x) ∣ S(x);A)p(S(x);I,A)p(I(x))).

*Spatial Regularisation*. Following the work of Kolmogorov and Boykov [28, 30], the smoothness term *E*_*F*_ of the energy function is defined from a Finsler metric. These authors decomposed the energy into *E*_*R*_ and *E*_*f*_ with weights *λ*_1_, *λ*_2_ ∈ *R*; that is,

(11) EF(S)=λ1ER(S)+λ2Ef(S).

The first part minimises the segmentation surface by a Riemannian metric, and the second one takes into account the orientation of the segmentation surface in the metric. We consider that the isotropic Riemannian metric from the image is defined by *D*(*x*) = *g*(||∇*I*(*x*)||)*I*, where *I* is an identity matrix, *g*(*x*) = (exp⁡(−*x*/*γ*))^1/3^, and *γ* is estimated as the average of ||∇*I*(*x*)||. The pairwise potentials are defined by

(12) ψxy(S(x),S(y);I) =λ1ωxR(y)(1−S(x))S(y)  +λ2ωxf(y)(S(x)(1−S(y))−S(y)(1−S(x))),

where *ω*_*x*_^*R*^(*y*) = *g*(||∇*I*(*x*)||)/||*x* − *y*|| and *ω*_*x*_^*f*^(*y*) is assigned by the dot product between ∇*I*(*x*) and the vector defined by *x* and *y*.

Therefore, the proposed model is characterised by a pseudo-Boolean function defined on unary and pairwise potentials, and the optimal labelling is determined by applying the min-cut/max-flow algorithm of [31].

### 2.3. Obtaining the Coarse Segmentation

The coarse segmentation *S*_*C*_ is used to define the domain of nonrigid registrations and similarity measures of the atlas selection. *S*_*C*_ is obtained using a probabilistic atlas. We observed that *S*_*C*_ improves the performances of the multiatlas segmentation with respect to the initial solution, *S* ^0^, or the conventional approach (i.e., without defining the domain using binary masks for registering and atlas selections) [32].

In a probabilistic atlas, the information from atlases is usually combined into a generative model in a common coordinate system. In this paper, an atlas is selected as a reference to which all atlases are then coregistered using a particular registration method [17]. An affine registration is applied for spatial normalisation. The principle that we have adopted for spatially normalising transformations is that these transformations should align the anatomical structures with low computational effort. We only expect a coarse segmentation of the target image that improves the results of the multiatlas segmentation. For this purpose, the affine transformations are sufficient. In general, the affine registration is performed with intensity-based similarity measures. Because we have an initial solution (*S* ^0^) and because iterative segmentation-registration approaches perform better than a single propagation of the probabilistic atlas [20], we use a fast affine registration based on the alignment of the labelled images, where its parameters are calculated using the geometric moments of the labelled images [33, 34]. The atlases are coregistered using the atlas-labelled images. A manual segmentation is selected as a reference, and the atlases are coregistered. Next, the statistical parameter spatial maps of the probabilistic atlas are calculated using (7) and (9).

Due to the complex dependencies between the unknown segmentation, the target image, and the registration parameters, this problem is simplified using an EM framework [35]. The E-Step captures the posterior probability of the structure depending on the registration, and the M-Step updates the registration parameters [20]. A coarse segmentation is obtained using an iterative method of registration-segmentation. Given an initial solution of the ROI (*S* ^0^) belonging to the target image, {*I*, *S* ^0^} are aligned into the selected atlas as a reference using the same affine transformation method that was used to construct the probabilistic atlas. Although the atlases are coregistered into the target image in the segmentation method (see Section 2.2), there is no loss of generality in assuming that {*I*, *S*_*k*_} are aligned into the normalised atlases using affine transformations. A segmentation of *I* is computed by minimising our energy function in (2), which is based on the statistical parameter spatial maps of the probabilistic atlas. The resulting segmentation is again used for registering {*I*, *S* ^1^} into the selected atlas as a reference.

This process converges with a few iterations. In each iteration, the centroid and the axes of the structure to be segmented are better estimated, and thus, the registration between {*I*, *S* ^*k*^} and the coregistered atlases is less biased, which also implies an improvement in the segmentation. Certainly, there is a feedback effect between the affine registration and the labelling using the minimisation of the energy function. Finally, an inverse affine transformation is applied to return the segmentation to the native space of the target image.

### 2.4. Multiatlas Segmentation

Given the coarse segmentation, a multiatlas approach improves the segmentation with higher accuracy. Not all of the atlases need to be registered into the ROI of the target image [9, 12]. An atlas selection framework is required to select the atlases that best propagate their labels. Several methods to rank atlases have been tested [9, 36]. These methods are generally based on a similarity measure between each atlas with respect to the target image. Other criteria, which are not based on the similarity between images, are not considered in this study (e.g., the age of the patients in the medical image analysis). The best strategy as a trade-off between reliability and computational cost consists of registering the target image into a reference in which the atlases were previously registered. This approach requires only one registration during runtime. Most often, the ranking is performed using an intensity-based similarity measure computed between the target image and each atlas. We propose using the DICE coefficient [37] as a similarity measure between an aligned coarse segmentation of the target image and each aligned atlas-labelled image. The above affine registration, which is based on geometric moments of the labelled images, is used for the alignment of images because it was previously used for calculating the coarse segmentation and because it is now also employed for the atlas selection.

After the atlases are ranked, the number of the selected atlases is required. Aljabar et al. [9] showed that, given an ordered list of atlases, the accuracy of the final segmentation rapidly increases to a maximum level followed by a gradual decline according to the number of fused atlases. This result indicates that a fixed number of atlases can be determined for each application.

*Nonrigid Registration*. We have chosen a technique based on the maximisation of an intensity-based similarity measure in combination with a deformation field parameterised by cubic B-splines [38]. Klein et al. [39] demonstrated that for some intensity-based similarity measures, the optimisation converges to the solution when a very small number of random samples of intensity pairs are used. The flexibility of the control point grid also allows for the introduction of a binary mask image, in which only the nonrigid transformation is applied. Here, this binary mask is defined using a dilated version of the coarse segmentation, *S*_*C*_. The dilation is used to include borders of the anatomical structure and of some surrounding tissues.

Given a target image, the selected atlases are first aligned into the target image using the above affine registration. These affine parameters are calculated with the coarse segmentation of *I*, *S*_*C*_, and the corresponding atlas-labelled images. This step is faster than the rest of the nonrigid registration. Next, a multiresolution scheme is used in the nonrigid registration step to avoid local minima. 

*Label Fusion Method*. Several methods have been proposed to combine the propagation segmentations of the selected atlases into a single segmentation. These methods include the majority voting rule [11], STAPLE [22], or the minimisation of an energy function with intensity and prior terms [23]. In this work, we consider a segmentation method that uses graph cuts to optimise a discrete energy function. Given the registered atlases to the target image, a statistical model of appearance and shape is computed with (7) and (9). Subsequently, a graph-cut technique is used to minimise the energy function defined in (2), and the consensual segmentation is obtained from the graph cut [23]. The difference with the segmentation based on the proposed probabilistic atlas is that the fusion method does not require iterations of registration-segmentation. Now, the statistical parameter spatial maps are not biased because the selected atlases are considered to be better registered into the target image. Therefore, given the set of selected registered atlases into the target image, the statistical parameter spatial maps are calculated, and a consensual segmentation is obtained by S* = argmin⁡_*S*_(*E*_*B*_^*A*′^(*S*) + *E*_*F*_(*S*)) using graph cuts.

## 3. Experiments with Liver CT Data

The atlases and the test data were taken from a public database for liver segmentation [40] (http://www.sliver07.org/). We used this public database because it allows us to compare our approach to a large number of other segmentation methods, including other atlas-based schemes [32]. A total of 30 images were randomly divided into 20 images that were used for the training set, and the remaining 10 images were used for the test set. All of the CT images are enhanced with a contrast agent and scanned in the central venous phase using diverse scanners (machines with 4, 16, and 64 detector rows). The pixel spacing varies from 0.55 to 0.88 mm, and the interslice distance varies from 1 to 3 mm. Most images in this study exhibited pathologies, including tumours, metastasis, and cysts of different sizes.

To evaluate the quality of a given segmentation, we used the following five error measures [40]: overlap error (*m*_1_), relative absolute surface difference (*m*_2_), average symmetric distance (*m*_3_), root mean square symmetric distance (*m*_4_), and maximum symmetric distance (*m*_5_). The main advantage of using multiple measures rather than a single measure is that different measures detect various aspects of the segmentation quality. The problem of how to combine the different measures to produce a ranking of the segmentation results is solved by transforming the result of each error measure to a common scale and averaging the resulting values to obtain the final score [40]. Each measure is converted to a scale ranging from 0 to 100 by

(13) αj(X,Y)=max⁡(100−25mj(X,Y)m~j,0), j=1,…,5,

where *X* and *Y* represent the manual and automatic segmentation binary images, respectively, *m*_*j*_ is an error measure, and ${\tilde{m}}_{j}$ is the corresponding reference value, which was obtained by averaging the manual segmentations. A score of one hundred points (*α*_*j*_ = 100) is a perfect match with the reference segmentation, and a score around 75 is equivalent to human performance. The final score is the average of the individual measure scores: *α*(*X*, *Y*) = ∑_*j*=1_^*j*=5^0.2 · *α*_*j*_(*X*, *Y*). To compare our approach with other methods and applications, the resulting segmentations were also measured using the DICE coefficient.

### 3.1. Experiments with Ground Truth

The training atlases are used for experimental validation in a leave-one-out fashion: one atlas is used as the target image, and the other 19 are used as training atlases. The manual segmentation of the target image is used as the ground truth. This procedure is repeated in all atlases that belong to the training set.

For the coarse segmentation and the atlas selection, the atlases and the target image are first subsampled by a factor of two in each dimension to reduce the computation time. Preliminary experiments showed that using the full-resolution data increased the computation times and negligibly improved the results. However, the nonrigid registrations of the atlases into the target image are performed within the original resolution.

#### 3.1.1. Setting the Probabilistic Atlas Parameters through Training

**Figure 2:**
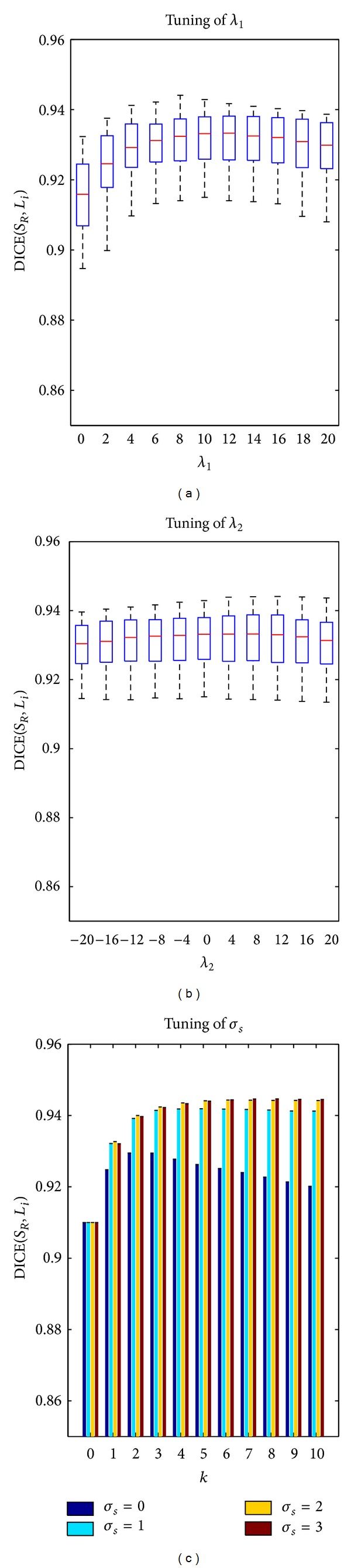
Tuning of parameters *λ*_1_, *λ*_2_, and *σ*_*s*_. (a) The distributions of DICE(*S*_*R*_, *L*_*i*_) with the values of *λ*_1_, where *S*_*R*_ is the reference segmentation and *L*_*i*_ is the automatic segmentation obtained with *λ*_1_ (*σ*_*s*_ = 1, *k* = 1, *λ*_2_ = 0). (b) The distributions of DICE(*S*_*R*_, *L*_*i*_) with the values of *λ*_2_ (*σ*_*s*_ = 1, *k* = 1, *λ*_1_ = 10). (c) The average DICE in segmenting all target images for *σ*_*s*_ and for each iteration (*λ*_1_ = 10, *λ*_2_ = 4).

Three parameters of the probabilistic atlas are tuned: (a) the scale parameter *σ*_*s*_ of (7), (b) the multipliers *λ*_1_ and *λ*_2_ of the energy function, and (c) the number *k* of iterations in (1). Twenty leave-one-out segmentations on the training atlases are performed to determine the tunable parameters. These parameters are varied in certain ranges, and their effects are measured by the overlap between the resulting segmentation and the ground truth. The DICE coefficient is selected as the measure of the segmentation overlaps. In our experiments, *k* varies from 1 to 10. Because *σ*_*s*_ and the multipliers are coupled together, an iterative adjustment is used. We observed that the Riemannian metric is more influential than the surface orientation term in the optimisation process. Therefore, *λ*_1_ is tuned first, and then *λ*_2_ is adjusted. Considering 3D grid graphs with a 6-neighborhood system in the CRF model, *σ*_*s*_ = 1, *k* = 1, *λ*_2_ = 0, and ∇*I*(*x*) are calculated by Gaussian derivatives at a scale of 1; *λ*_1_ is varied, and DICE is used to detect the optimal value (see Figure 2(a)), which is tuned with *λ*_1_ = 10. With a 6-neighborhood system, ∇*I*(*x*) is easily decomposed into *ω*_*x*_^*f*^(*y*) and *λ*_2_ can take positive or negative values due to the orientation of ∇*I*(*x*). Indeed, the grey level of the liver may be brighter than in other adjacent tissues (the majority of the time), but it may also become darker in other areas (e.g., liver-kidney contact).  Figure 2(b) presents the trend of *λ*_2_ (*σ*_*s*_ = 1, *k* = 1, *λ*_1_ = 10), and the flow term shows the least impact on the success of the segmentation. With fixed values of *λ*_1_ = 10 and *λ*_2_ = 4, *σ*_*s*_ and *k* are tuned to 2 and 4, respectively (Figure 2(c)). The tuning process is repeated with *σ*_*s*_ = 2. The parameters did not vary substantially (*λ*_1_ = 8, *λ*_2_ = 4), and the performances were virtually the same.

The experimental results show the utility of performing an iterative scheme of segmentation-registration for obtaining *S*_*C*_ (see Figure 2(c)). Compared to the classical registration-segmentation with a probabilistic atlas (*k* = 1), the iterative method given in (1) performs better for *k* > 1 due to the more reliable estimation of the posterior probabilities, which are produced by a better registration, between the probabilistic atlas and the target image. We also observe the effect of spatial regularisation {*μ*(*x*, *l*), *σ* ^2^(*x*, *l*)}_*l*∈{0,1},*x*∈*Ω*_ref__ of *A* by linear diffusion. With the linear diffusion, the parameter estimations of the appearance model are less biased and the iterative process is convergent and stable, otherwise (*σ*_*s*_ = 0) it is not.

Furthermore, we have experimentally observed that if our probabilistic atlas does not have the appearance model as in [15], the numerical scheme does not work because there is a weak feedback effect between segmentation and registration. Indeed, the intensities of the target image are only used in the regularisation term of the optimisation process; that is, the pairwise potentials are only updated in each iteration of the EM framework. Therefore, there are no substantial changes in the new segmentation, and, consequently, the parameters of the affine registration are not varied.

#### 3.1.2. The Ranking of Atlases for the Multiatlas Segmentation

Any ranking of atlases based on similarity measures requires a spatial normalisation step. In this paper, the atlases and the target image are registered into a common reference using affine transformations. An atlas of the training set is chosen as a reference for the spatial normalisation [41].

Three criteria for ranking the atlases are tested: (a) a random order that does not require any registration task, (b) using Mutual Information (MI) as in [9], and (c) our method based on a similarity measure between each atlas-labelled image and *S*_*C*_ with DICE coefficient. After the atlases are ranked, they are registered into the target image. The same nonrigid registration method is applied for the three ranking criteria. All nonrigid registrations are computed using* Elastix* [42], which is an available public package for medical image registration. This framework is based on the techniques described in [38, 39, 43]. We employ a four-level multiresolution scheme. In our experiments, the B-Spline grid spacings are 64, 32, 16, and 8 mm in all directions for the four respective resolutions. The negative MI is used as the cost function, which is implemented according to [43]. For the optimisation of the cost function, an iterative stochastic gradient descent optimiser is used [39]. In each iteration, 2000 random samples are used to calculate the derivative of the cost function. Random samples are acquired from a domain defined by a binary mask, which is set by a dilated version of *S*_*C*_.

**Figure 3:**
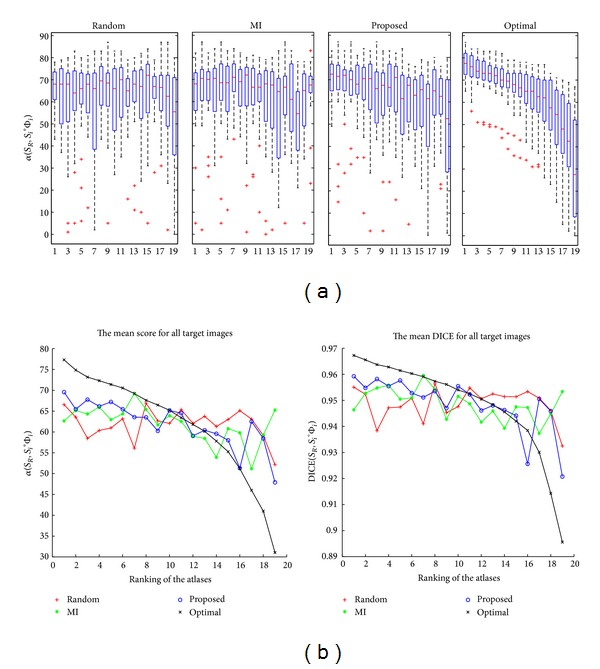
Relationship between the individual atlases and their performance in segmenting the target image. The upper part of the graph illustrates the scoring distribution in segmenting by label propagation for a given rank (random, MI, proposed, or optimal) and for an individual atlas, $\alpha (S_{R},{\tilde{S}}_{i})$. The lower part of the figure shows the average scoring and the average DICE on all target images against the rank.

We denote by Φ_*i*_ the combined transformation of the affine and nonrigid registrations from the domain of *I* to *A*_*i*_.  Figure 3 shows the relationship between the individual atlases and their performance in segmenting the target images. The results are shown in two graphs: (a) the distributions of $\alpha (S_{R},{\tilde{S}}_{i})$ for a given rank (random, MI, proposed, or optimal), where *S*_*R*_ is the ground-truth segmentation of the target image, *i* is the order of atlas in the database from the similarity to the target image, and ${\tilde{S}}_{i}=S_{i}\circ \Phi_{i}$ is the corresponding deformed labelled image and (b) the average of $\alpha (S_{R},{\tilde{S}}_{i})$ or $\text{DICE}(S_{R},{\tilde{S}}_{i})$ against the rank where each plotted point shows the average score or DICE for all of the target images at a given rank. The optimal ranking is obtained by sorting in descending order according to the score of each label using *S*_*R*_. Our atlas selection is the most similar to the optimal ranking. Although the segmentations are not fused in this experiment, there are relationships between the atlas rankings and the segmentation accuracies derived from fusing these selected atlases (which will be shown in the following experiment).

#### 3.1.3. Stopping Criterion

**Figure 4:**
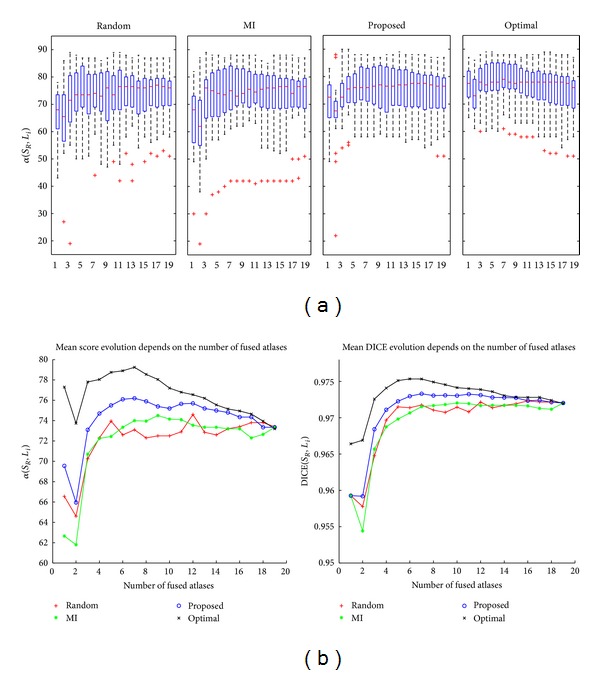
Relationship between the segmentation accuracy and the number of fused atlases. The upper part of the graph illustrates the scoring distribution in segmenting by label propagation for a number of fused atlases depending on the type of rankings, *α*(*S*_*R*_, *L*_*i*_). The lower part of the figure shows the mean scoring and DICE in all target images against the different number of fused atlases and for different atlas selection criteria.

The following experiment describes a test of the effect of varying the number of selected atlases for generating the consensual segmentation. Given a rank of atlases and a number of selected atlases, the single consensual segmentation is computed. The same method of nonrigid registration and label fusion are applied for all samples and for all rankings. The registrations are performed under the same conditions as in the previous experiment. The label fusion method is based on minimising our energy function, as explained in Section  2.4.  Figure 4 shows how the segmentation accuracy varies with the number of fused atlases. In the upper part of the figure, each box and whiskers in the upper part of the graph illustrate the distributions of *α*(*S*_*R*_, *L*_*i*_) for each criterion depending on the ranking of atlases. *L*_*i*_ is a binary image of the target image obtained by the fusion method with the first *i* selected atlases. In the lower part of the figure, each plotted point shows the average score or DICE coefficient in segmenting all of the target images for the number of fused atlases. The general pattern shows a sharp initial increase up to a maximum score followed by a gradual decline, as in [9]. The decrease in the score when two atlases are fused is due to increased uncertainty. Discrimination with only two registered atlases cannot rely on other sources in doubtful cases. We also observe that the score system is more sensitive than the DICE coefficient. The DICE coefficient does not exhibit a decrease when two atlases are fused.

Our approach requires 6 or 7 atlases for obtaining the maximum score and DICE (*α* = 76.3 ± 8.6 and DICE = 0.973 ± 0.007). The other atlas selections (MI and random) require a greater number of atlases for obtaining the maximum score (MI: 9, random: 12), and their scores are worse (MI: *α* = 74.50 ± 10.9 and DICE = 0.972 ± 0.01, random: *α* = 74.80 ± 9.5 and DICE = 0.972 ± 0.008). However, no significant improvements are obtained between our proposed method and the other atlas selections. Only the effect of atlas selection was evaluated. Recall that the methods of registration and fusion are applied in the same manner in all atlas selection frameworks. Statistical significance is evaluated using a paired two-sided Wilcoxon test, where a *P* value of <0.05 indicates significant improvement. Given a fixed number of atlases equal to 6 and compared to our proposal, the *P* values are 0.456 and 0.297 for the MI-based and random-based atlas selections, respectively.

#### 3.1.4. Comparison with a Conventional Multiatlas Segmentation

**Figure 5:**
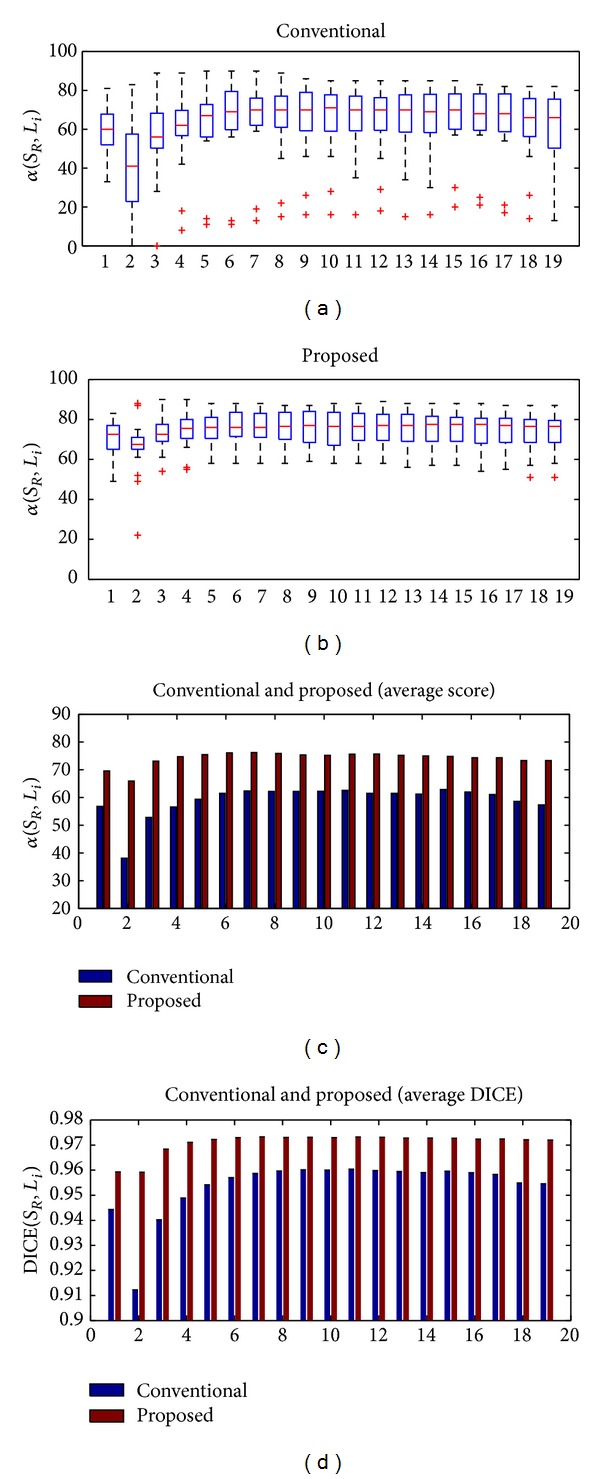
Comparison between a conventional multiatlas segmentation and the proposed approach depending on the number of fused atlases. (a) The distribution of *α*(*S*_*R*_, *L*_*i*_) with *S*_*R*_ is the ground-truth segmentation, and *L*_*i*_ is the consensual segmentation with the first *i* atlases. (b) The distributions of *α*(*S*_*R*_, *L*_*i*_) for the proposed approach. (c) The average scores in segmenting all target images for the number of fused atlases and for each approach. (d) The average DICE in segmenting all target images for the number of fused atlases and for each approach.

The atlas database from Heimann et al. [1] is used for comparing several methods for liver segmentation from CT images. One of the presented methods is based on multiatlas segmentation [32]. A fixed number of twelve selected atlases are registered to the target image using an affine transformation followed by a B-spline approach with multiple resolutions. The cost function used for the registrations is the negative MI. Using this approach, we implement a conventional multiatlas segmentation. The atlases are registered to the entire target image as in [32], and the ranking of the atlases is performed according to [9]. We compare this approach with our proposed approach. Our proposed label fusion is used in both approaches. The upper part of Figure 5 illustrates the distributions of *α*(*S*_*R*_, *L*_*i*_) with the number of fused atlases. The lower part of the figure shows the average scores and DICE in segmenting all of the target images for the number of fused atlases and for each approach. Setting the number of fused atlases to 6, the conventional approach provides poorer results (*α* : 61.5 ± 25.1, DICE : 0.957 ± 0.037) than the proposed approach (*α* : 76.3 ± 8.6, DICE : 0.973 ± 0.007). For all cases, the *P* values are always less than 0.05, indicating a significant improvement between our approach and the conventional one for any number of fused atlases.

### 3.2. Results

**Figure 6:**
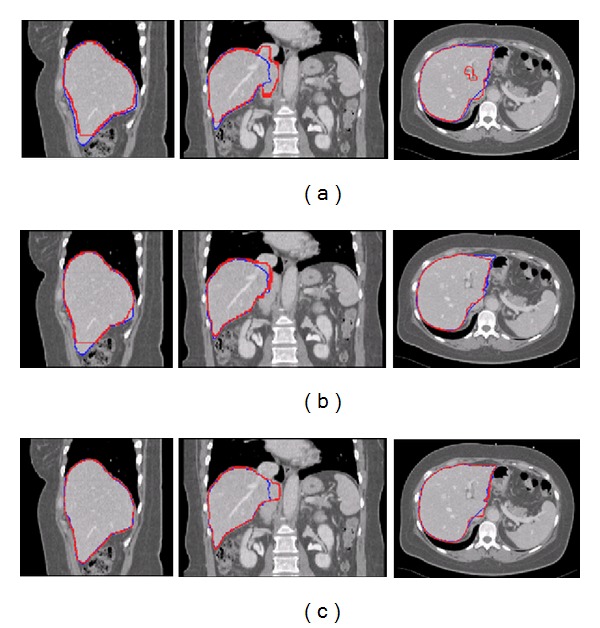
Resulting segmentations for a representative patient from the training database. From left to right, a sagittal, coronal, and axial slice for the (a) initial solution, (b) coarse segmentation, and (c) multiatlas segmentation. The outline of the ground-truth segmentation is in blue; the outline of the segmentation of the method described in this paper is in red.

The performances of the three stages (initial solution, coarse segmentation, and label propagations) are evaluated by computing {*m*_*j*_, *α*_*j*_}_*j*=1,…,5_ for the case of liver segmentation.  Table 1 presents the mean values and standard deviations for each step of the measures and scores of the segmentations of the target images. The DICE coefficients are 0.910 ± 0.024, 0.943 ± 0.016, and 0.973 ± 0.007 for the initial solution, coarse segmentation, and multiatlas segmentation method, respectively, and the scores are 34.4 ± 14.3, 56.3 ± 15.1, and 76.3 ± 8.6 (see Figure 6). The evaluation of the test images is performed by an external team by submitting the results to the website as in [1]. The scores of the test images are lower than that of the training set: 70.5 ± 16.4 (test), 76.3 ± 8.6 (training).

**Figure 7:**
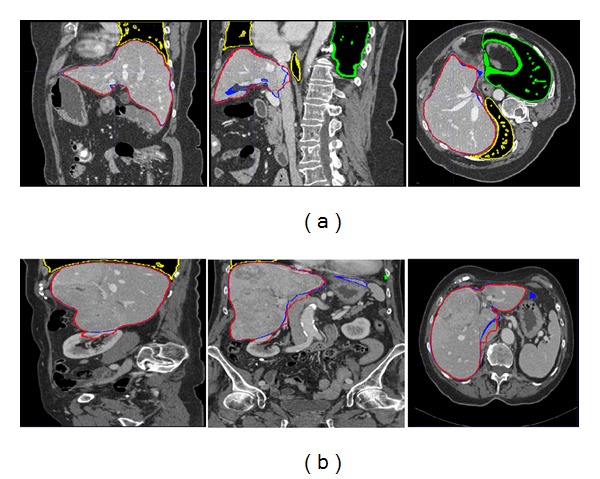
From left to right, a sagittal, coronal, and axial slice for an easy case (a) and for a difficult case (b). The outline of the ground-truth segmentation is in blue, and the outline of the segmentation of the method described in this paper is in red. The outline of the lung segmentations is also illustrated in green (left lung) and yellow (right lung).

**Figure 8:**
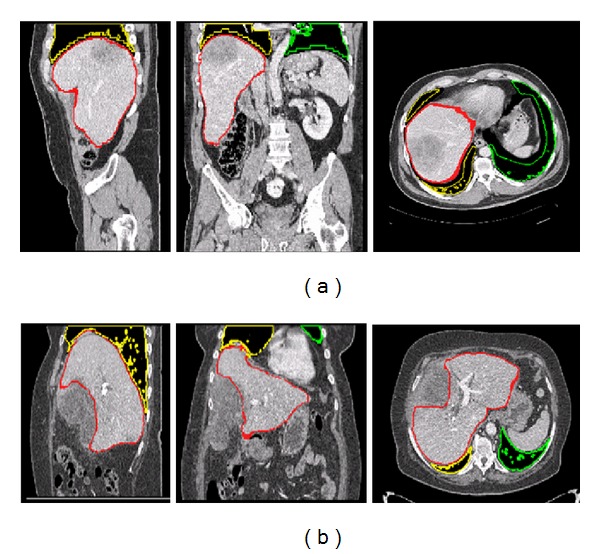
Large tumours in the boundary of the livers from the test database. From left to right, a sagittal, coronal, and axial slice for the two worst cases, which present the worst scores. The outline of the ground-truth segmentation is in blue, and the outline of the segmentation of the method described in this paper is in red. The outline of the lung segmentations is also illustrated in green (left lung) and yellow (right lung).

Figure 7 shows slices from two cases, drawing the result of the method (in red) and the manual segmentation (in blue) from the training database.  Figure 8 shows two cases with tumours from the test database. The proposed approach is shown to be robust for the first case but not for the second case when tumours appear in the boundary of the liver.  Figure 8(b) shows the worst score of all images. The results listed at http://www.sliver07.org/ show that our approach is comparable to the performance of human experts and other recently published results.

The method is implemented with the ITK library, and some procedures are parallelised with OpenMP. The major computational cost is clearly the multiatlas segmentation method. However, the low cost of multicore processors is making this approach faster. The average computational times are 17.16 ± 6.18 s and 33.29 ± 9.27 s for the initial solution and coarse segmentation (including the initial solution), respectively. The average computational time for registering one atlas is 34.63 ± 2.93 s. When fusing six atlases into the target image, the total runtime to segment one sample is 261.35 ± 18.6 s ([Dual CPU] Intel Xeon E5520 @ 2.27 GHz).

## 4. Discussion and Conclusions

An atlas-based segmentation framework is proposed that combines low-level operations and a fast probabilistic atlas with multiatlas segmentation. The proposed combination provides high accuracy in segmentation due to registrations and atlas selection based on ROIs and coarse segmentations. Our approach shares the following common elements between the proposed probabilistic atlas and the multiatlas segmentation: (a) the spatial normalisation and (b) the segmentation method. Spatial normalisation is used for both constructing the probabilistic atlas and for obtaining the atlas selection for each ROI. The segmentation method is based on standard CRF models, allowing for the incorporation of appearance and shape in a single unified manner.

Specifically, ROIs of the target image are obtained using low-level operations. A probabilistic atlas is constructed for each ROI using affine registrations based on geometric moments from labelled images. The label prior probability was estimated by voting, and the image likelihood was modelled using independent Gaussian distributions for each pixel and for each label. To overcome bias in the statistical parameter spatial maps of the probabilistic atlas, they were smoothed using spatial linear diffusion. Other statistical models can be used as in [23], where the image likelihood is approximated using a Parzen window estimator. An advantage of our approach is that there is only one probabilistic atlas; it is not necessary to recalculate the statistical parameter spatial maps for each target image.

Given an initial solution in a ROI using low-level operations, an EM framework is used to obtain a coarse segmentation. In each iteration, a new segmentation is computed by minimising a discrete energy function, and this labelling is again aligned with the probabilistic atlas by geometric moments belonging to labelled images. The energy function, which is applied for segmentation, combines the maximisation of the posterior probability and the minimal area of the separation surfaces between the object and background under an image-based Finsler metric. The resulting energy function was globally minimised using graph cuts. A few iterations were sufficient for this process to reach convergence. We experimentally observed that the iterative method outperforms the classical approach of a single registration and obtaining the segmentation using the probabilistic atlas. We also observed that the effect of spatial regularisation of the statistical parameter spatial maps of the probabilistic atlas by linear diffusion causes the process to converge within a few iterations.

The computational time for obtaining the coarse segmentation is low due to affine registrations based on the geometric moments of the labelled images and the min-cut/max-flow algorithm.

The coarse segmentation is employed in the next step to define the registering mask and to obtain the rankings of atlases that are more similar to the target image for each ROI. Three aspects of the multiatlas approach were analysed: image registration, atlas selection, and label fusion methods. In image registration, we proposed aligning the selected atlases to the target image by geometrical moments using *S*_*C*_ followed by a local deformation in the ROI. For atlas selection, the DICE coefficient was used to rank the atlases. Different atlas selection methods were compared. Although our atlas selection framework is the most similar to the optimum one and provides better segmentation results, the improvements are not statistically significant with respect to the other atlas selections. However, our complete approach shows significant improvements relative to the conventional framework for any number of fused atlases in liver segmentation. In label fusion, a standard CRF model is used for MAP inference.

Comparing the segmentation results between different published methods is always difficult. The quality of the databases used for validation, the anatomical definition of the structure, the quality of expert segmentations, the populations studied, and the different measures reported all make it difficult to compare results. With these caveats in mind, we compared our segmentation results with other approaches that used the same database, that is, http://www.sliver07.org/ [40]. Maklad et al. [44] used blood vessel information to segment the liver through the portal phase of an abdominal CT dataset. This semiautomatic method requires a small group of manual seeds. Their results are evaluated with an overall score of 85.7, which is ranked as the best in this public database. Peng et al. [8] presented another semiautomatic method, which is a level set-based variational approach. Their model is not restricted by training data and can be applied to livers of any shape. They reported a score of 80 ± 4. Ruskó et al. [3] proposed a fully automatic method for liver segmentation. Their method is essentially an advanced region growing and performs with an average total score of 61 ± 21. Linguraru et al. [45] presented an automated segmentation of livers. An affine invariant shape parameterisation is combined with a geodesic active contour and graph cuts. They reported a score of 76 ± 6. Our method has higher scores than the other automatic methods and close to those of the best semiautomatic methods. Our results provide high accuracy in automatic segmentation, and the computational time depends on the level of accuracy requested by the user. The results listed at http://www.sliver07.org/ show that our approach is comparable to that of human experts and other recently published results.

However, the issue of liver segmentation has only a single ROI and does not show the full potential of the proposed method. New problems that require two or more regions of interest should be analysed. We have applied the label fusion method to the hippocampal segmentation from magnetic resonance imaging [46]. It is partially shown that the proposed methods in this work are generic and could be incorporated to other applications.

## Figures and Tables

**Table 1:** Average values of the measures and scores for all 20 training images and 10 test images: volumetric overlap error (*m*_1_), relative absolute volume difference (*m*_2_), average symmetric surface distance (*m*_3_), root mean square symmetric surface distance (*m*_4_), and maximum symmetric surface distance (*m*_5_).

Type		*m*_1_ [%]	*m*_2_ [%]	*m*_3_ [mm]	*m*_4_ [mm]	*m*_5_ [mm]	*α*()
Initial Solution	Measures	16.5 ± 3.8	8.6 ± 5.8	3.6 ± 1.6	7.1 ± 3.9	50.2 ± 24.8	
	Scores	37	56	21	19	39	34.4

Affine prob. Atlas	Measures	11.2 ± 2.8	2.3 ± 1.8	2.2 ± 0.9	4.4 ± 1.9	35.8 ± 15.5	
	Scores	56	88	44	40	53	56.3

The proposed approach 20 training images	Measures	5.2 ± 1.2	0.9 ± 1.2	1.0 ± 0.4	2.2 ± 1.1	26.9 ± 10.7	
	Scores	79	91	76	70	65	76.3

The proposed approach 10 training images	Measures	7.6 ± 3.2	−0.5 ± 3.9	1.3 ± 0.7	2.9 ± 1.8	24.7 ± 10.7	
	Scores	70	87	68	60	68	70.5
